# Efficacy and safety of GLP-1 receptor agonists in the treatment of obese patients with chronic heart failure: a meta-analysis

**DOI:** 10.3389/fcvm.2025.1633114

**Published:** 2025-10-03

**Authors:** Anna Jia, Ming Yang, Tianhong Wang, Yusi Hua, Huimin Lu

**Affiliations:** ^1^ Department of Anesthesiology, West China Hospital, Sichuan University, Chengdu, China; ^2^ West China School of Nursing, Sichuan University, Chengdu, China; ^3^ First Clinical Medical College, Shandong University of Traditional Chinese Medicine, Jinan, China; ^4^ The Department of Clinical Research, West China Hospital, Sichuan University, Chengdu, China; ^5^ Center of Excellence for Pancreatitis, Institute of Integrated Traditional Chinese and Western Medicine, West China Hospital, Sichuan University, Chengdu, China

**Keywords:** GLP-1 receptor agonists, chronic heart failure, obesity, meta-analysis, semaglutide

## Abstract

**Objective:**

To investigate the efficacy and safety of Glucagon-Like Peptide-1 Receptor Agonists(GLP-1RAs) (Liraglutide, Semaglutide, Exenatide, Dulaglutide, Lixisenatide, and Tirzepatide) in obese patients with chronic heart failure (CHF).

**Method:**

A systematic search was performed in 3 databases (Pubmed, Embase, and Cochrane Library) for articles evaluating the effectiveness and safety of GLP-1RAs (Liraglutide, Semaglutide, Exenatide, Dulaglutide, Lixisenatide, and Tirzepatide) for the treatment of obese patients with CHF from the time the database was created until 5 January 2025. Meta-analyses were performed to evaluate: primary outcomes, including all-cause mortality, cardiovascular mortality, and worsening heart failure events; secondary outcomes, encompassing changes in body weight, Kansas City Cardiomyopathy Questionnaire Clinical Summary Score (KCCQ-CSS), 6-minute walk distance, B-type Natriuretic Peptide (BNP) level, high-sensitivity C-Reactive Protein (hs-CRP) level, and left ventricular ejection fraction (LVEF) level; and safety outcomes, specifically gastrointestinal adverse events and serious adverse events.

**Results:**

A total of 6 papers were included for Meta-analysis. The primary clinical outcomes: all-cause mortality [OR=0.89, 95% confidence interval (CI): 0.40–2.00, *p* = 0.78], cardiovascular mortality (OR = 0.93, 95% CI: 0.22–4.00, *p* = 0.92) and worsening heart failure events (OR=0.43, 95% CI: 0.30–0.59, *p* < 0.00001); For secondary outcomes, change in body weight (MD = −7.90, 95% CI: −15.44 to −0.35, *p* = 0.04), change in the KCCQ-CSS (MD = 6.81, 95% CI: 6.62–6.99, *p* < 0.00001),change in the 6-minute walk distance (MD = 15.91, 95% CI: 15.36–16.47, *p* < 0.00001), change in the BNP level (MD = −0.13, 95% CI: −0.21 to −0.05, *p* = 0.001), changes in the hs-CRP level (MD = −16.61, 95% CI: −48.53 to 15.31, *p* = 0.31) and change in the LVEF level (MD = −0.91, 95% CI: −2.12 to 0.29, *p* = 0.14). For safety outcomes, gastrointestinal adverse events (OR=0.87, 95% CI: 0.11–7.05, *p* = 0.90) and serious adverse events (OR=0.63, 95% CI: 0.37–1.08, *p* = 0.09).

**Conclusion:**

The study results show that GLP-1RAs significantly reduce the risk of worsening heart failure events and improve cardiac function, suggesting that GLP-1RAs are promising treatment options for obese patients with CHF.

## Introduction

Heart failure (HF) is a progressive clinical syndrome that occurs when the heart is unable to pump blood effectively enough to meet the body's oxygen needs. People with a clear diagnosis or progressive onset of HF symptoms are called chronic heart failure (CHF) (1). The increasing incidence and prevalence of CHF is one of the most pressing therapeutic challenges in today's clinical medicine (2, 3). Characterized by diastolic dysfunction, dyspnoea, and reduced exercise tolerance, CHF poses a growing global healthcare burden due to its limited therapeutic options, and poor prognosis (4, 5).

Obesity is a growing health problem worldwide and a significant risk factor for cardiovascular disease, especially CHF (6). Inflammation is a key factor contributing to cardiovascular injury (7). Obesity-driven systemic inflammation can cause coronary microvascular dysfunction and increased epicardial adipose tissue, leading to myocardial fibrosis and molecular alterations in cardiomyocytes, which ultimately triggers myocardial stiffness and diastolic dysfunction (8). In addition, obesity induces insulin resistance, which elevates blood pressure and promotes atherosclerosis, thereby impairing ventricular-vascular coupling, reducing exercise tolerance, and ultimately contributing to CHF (9). Currently, first-line treatment for CHF includes SGLT 2 inhibitors, *β* Beta-blockers, Mineralocorticoid Receptor Antagonist (MRA) and so on. A comprehensive treatment strategy for obesity-related CHF is lacking.

Glucagon-Like Peptide-1 Receptor Agonists(GLP-1RAs) can exert glucose-lowering and weight-loss effects by activating GLP-1 receptors (10). It is worth noting that, tirzepatide is a dual Glucose-dependent Insulinotropic Polypeptide (GIP) and GLP-1 receptor agonist that exerts synergistic effects by simultaneously activating GIP and GLP-1 receptors.In this study, we included it in the GLP-1RA category. Liu et al. (11) demonstrated that GLP-1RAs reduced weight in a nonlinear dose-response manner in obese or overweight (without diabetes) patients in a Meta-analysis of a randomized controlled trial. By targeting endothelial dysfunction, GLP-1RAs can improve microvascular function and vascular endothelial function (12). In the mouse model of Heart Failure with Preserved Ejection Fraction(HFpEF), the administration of GLP-1 RA Lira can alleviate cardiometabolic dysregulation, and improve the state of fibrosis and inflammation (13). Wong et al. (14) reported in a meta-analysis that GLP-1RAs improved cardiac function in type 2 diabetes patients, with liraglutide specifically increasing LVEF and reducing LVESV. Liu et al. (15) found that GLP-1RAs brought about clinical benefits by improving ventricular diastolic function (e. g., reducing filling pressure and promoting ventricular relaxation), especially for people without a history of heart failure in a meta-analysis of randomized controlled trials.Although GLP-1RAs have demonstrated multiple cardiovascular benefits in patients with type 2 diabetes and obesity, clinical efficacy and safety data on GLP-1RAs in patients with obesity combined with CHF remain limited.

This study aims to evaluate the clinical efficacy and safety of GLP-1RAs (Liraglutide, Semaglutide, Exenatide, Dulaglutide, Lixisenatide, and Tirzepatide) in obese patients with CHF through a systematic review and meta-analysis.

## Materials and methods

### Search strategy

This meta-analysis followed the 2020 guidelines developed by Preferred Reporting Project for Systematic Review and Meta-Analysis (PRISMA) (16). A comprehensive search was conducted in three databases, including PubMed, Embase, and Cochrane Library to retrieve the literature published as of January 5,2025. The search technique followed the PICOS principles and used a mixture of MeSH terms and unrestricted text phrases. The search method used was to combine the terms “Liraglutide”, “Semaglutide”, “Exenatide”, “Dulaglutide”, “Lixisenatide”, “Tirzepatide” and “chronic heart failure”. A detailed summary of the searched records is provided in Supplementary Material S1.

### Inclusion and exclusion criteria

The inclusion criteria are as follows: (1) age ≥18 years; (2) Patients diagnosed with CHF (17) (including HFpEF and HFrEF); (3) Obesity (with a body mass index of at least 30); (4)At least one patient cohort was treated with GLP-1RAs (liraglutide, semaglutide, exenatide, dulaglutide, lixisenatide or tirzepatide), with or without other treatments;(5) At least one of the following results was recorded: all-cause mortality events, cardiovascular mortality, worsening heart failure events, gastrointestinal adverse events, serious adverse events, change in body weight, change in the KCCQ-CSS, change in the 6-minute walk distance, change in the BNP level, change in the hs-CRP level and change in the LVEF level; (6) Study types: randomized controlled trials.

Exclusion criteria were as follows: (1) other types of articles, such as review, letter, conference, case reports, protocols, meeting, proceeding, abstract, meta-analysis, etc; (2) other diseases; (3) unrelated; (4) missing data; (5) cohort of repeat patients;(6) acute heart failure.

### Selection of studies

Literature screening, including elimination of duplicate entries, was performed using EndNote (version 20; Clarivate Analytics). Two independent reviewers separately conducted the first search, removed duplicates, evaluated the title and abstract to ensure their relevance, and finally read the full text to classify each study as inclusion or exclusion. The excluded studies and their potential bias introduced were discussed and consensus was reached through consultation. In the absence of consensus, a third reviewer acted as the mediator to determine the final number of literature included.

### Data extraction

Data were extracted independently by the two reviewers. The retrieved data include the following data: (1) the basic information of the study, such as the first author, year of publication, study method, sample size, and follow-up time; (2) Basic characteristics of the individuals participating in the study, such as the number of patients, age, gender, and BMI; (3) Outcome measures: all-cause mortality, cardiovascular mortality, worsening heart-failure events, gastrointestinal adverse events, serious adverse events, change in body weight, change in the KCCQ-CSS, change in the 6-minute walk distance, change in the BNP level, change in the hs-CRP level, change in the LVEF level. In case of ambiguity, consensus was reached by consulting the third investigator. In the included studies, seven cohorts of patients received GLP-1RAs such as Liraglutide, Tirzepatide and Semaglutide, and seven additional cohorts received Placebo or Glimepiride. Based on this, a meta-analysis was performed to compare the efficacy and safety of GLP-1RAs in obese patients with CHF.

### Quality assessment

Two independent reviewers assessed the quality of the included studies. Randomized controlled trials were assessed with the Cochrane Risk of Bias tool. Any discrepancies in the evaluations were resolved through group consensus.

### Statistical analysis

Analyses were performed using Review Manager version 5.3. The study used mean difference (MD) and 95% CI for comparison of continuous variables and odds ratio (OR) and 95% CI for comparison of dichotomous variables. Median and interquartile spacing of the continuity variables were converted to mean and standard deviation. Statistical heterogeneity among the studies included in the analysis was evaluated using Cochrane's Q test and I^2^ index. Considering that the studies included in the analysis were derived from the open literature, it was generally more reasonable to choose a random effects model. A *p*-value below 0.05 was considered statistically significant.

## Results

### Search results

**Figure 1:**
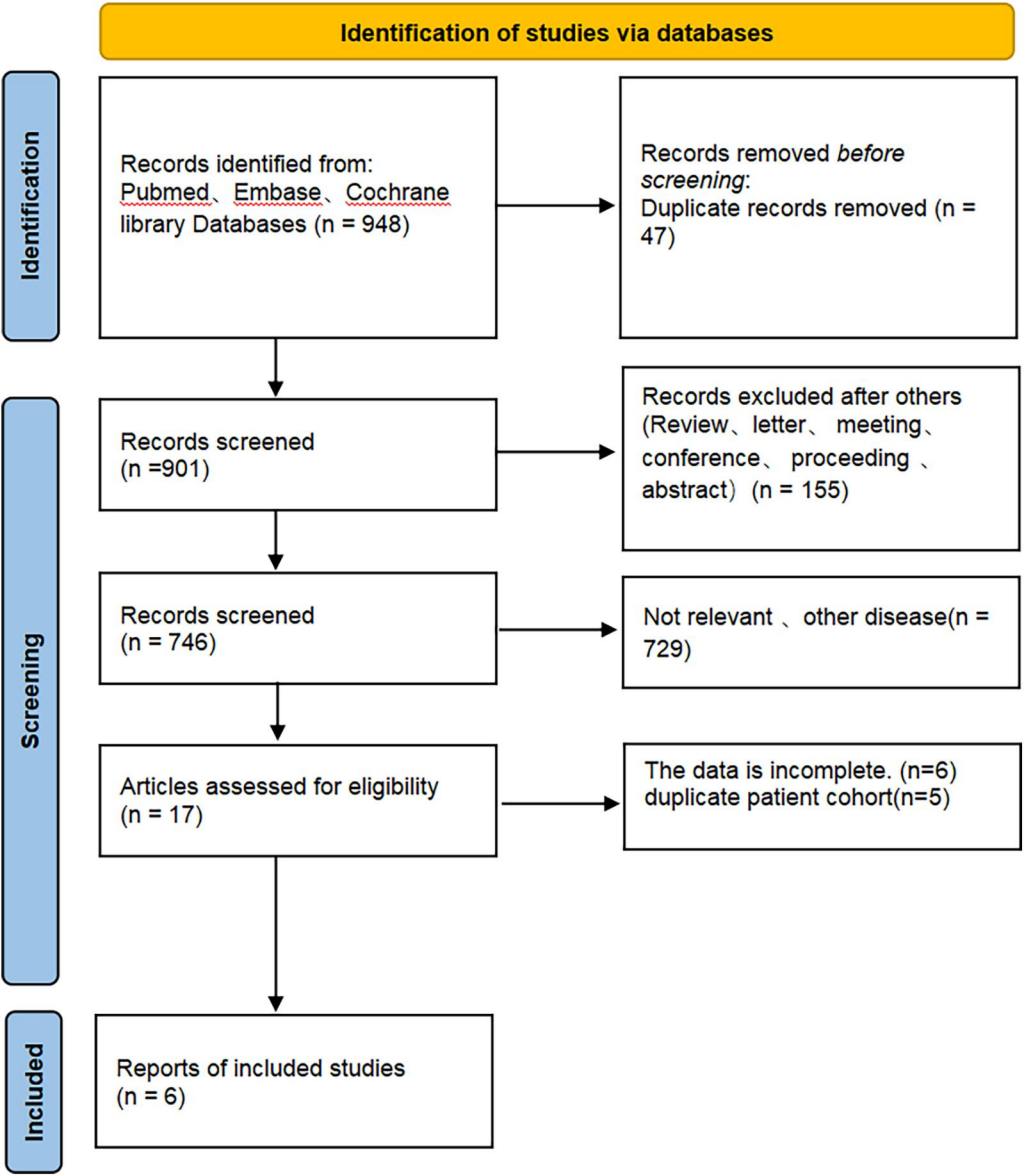
Flow chart of literature search strategies.

Figure 1 depicts the process of screening and integrating studies. A total of 948 studies were initially identified. A total of 901 articles remained after removing duplicate studies. A total of 884 articles were identified as unrelated when evaluating titles and abstracts. A total of 6 studies were selected for inclusion in this meta-analysis after a thorough examination of the full text.

### Patient characteristics and quality assessment

**Figure 2:**
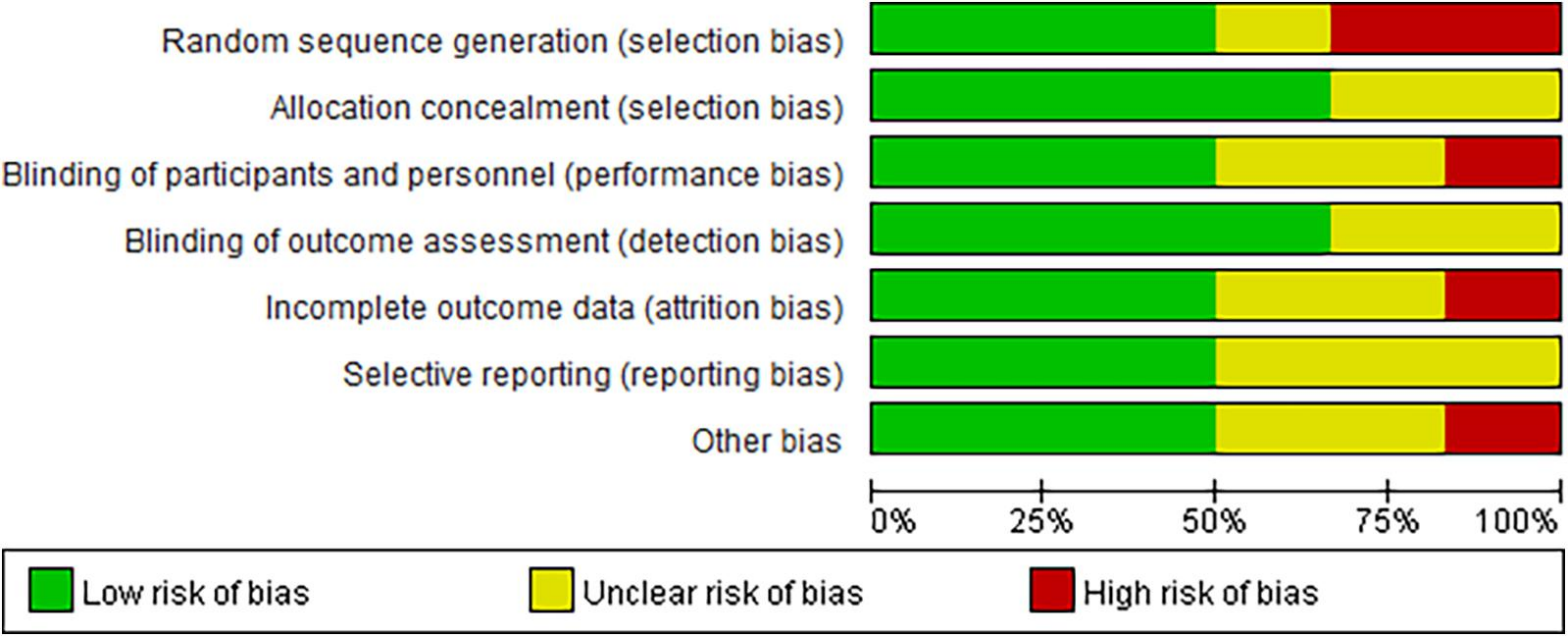
Risk of bias assessment of the included randomized controlled trials.

Six articles (18–23) were included in this meta-analysis, which consisted of seven randomized controlled trials. Detailed data on patient characteristics are presented in Table 1 and Figure 2 is the quality assessment results of the included studies.The meta-analysis focused on data from patients receiving GLP-1RAs to explore the efficacy and safety of GLP-1RAs in obese patients with CHF. More specifically, patients were divided into two groups according to their individual treatment regimen, one group received GLP-1RAs (Liraglutide, Tirzepatide, Semaglutide);the other group received placebo or Glimepiride. We performed a quality evaluation of included studies, and all articles were considered of good quality.

**Table 1:** The basic characteristics of included studies.

Author	Year	Study design	Treatment	Number	Age	M/F	BMI	Control	Number	Age	M/F	BMI	Follow-up	Outcomes
Michael R (18)	2024	RCT	Tirzepatide	364	65.5 ± 10.5	164/200	38.3 ± 6.4	Placebo	367	65.0 ± 10.9	174/193	38.2 ± 7.0	52weeks	all-cause mortality; cardiovascular mortality; Worsening heart-failure events
Milton (19)	2024	RCT	Tirzepatide	364	65.5 ± 10.5	164/200	38.3 ± 6.4	Placebo	367	65.0 ± 10.9	174/193	38.2 ± 7.0	52 weeks	Change in the KCCQ-CSS; Change in body weight; Change in the 6-minute walk distance; Change in the hs-CRP level; Change in the LVEF level;Serious adverse events
Javed (20)	2024	RCT	Semaglutide	573	70 (62–74)	296/277	37.0 (33.6–41.3)	Placebo	572	69.0 (62–75)	279/293	36.9 (33.4–41.5)	52weeks	all-cause mortality; cardiovascular mortality; Change in the KCCQ-CSS; Change in the 6-minute walk distance; Change in the hs-CRP level; Change in the BNP level
Morten (21)	2024	RCT	Semaglutide	406	NA	NA	NA	Placebo	379	NA	NA	NA	52 weeks	Worsening heart-failure events; Gastrointestinal adverse events; Serious adverse events
Morten (21)	2024	RCT	Semaglutide	167	NA	NA	NA	Placebo	193	NA	NA	NA	52 weeks	Worsening heart-failure events; Gastrointestinal adverse events; Serious adverse events
Thomas (22)	2017	RCT	Liraglutide	33	61 ± 7.6	24/9	30.5 ± 4.4	Glimepiride	29	63 ± 6.8	21/8	29.0 ± 3.2	18weeks	Change in the BNP level; Change in the LVEF level; Change in body weight
Anders (23)	2016	RCT	Liraglutide	122	65 ± 9.2	109/13	28.0 (3.8)	Placebo	119	65 ± 10.7	106/13	29.8 (4.6)	24weeks	Change in the 6-minute walk distance; Change in the BNP level; Change in the LVEF level

KCCQ-CSS, Kansas City Cardiomyopathy Questionnaire Clinical Summary Score; BNP, B-type natriuretic peptide; hs-CRP, high-sensitivity C-Reactive Protein; LVEF, left ventricular ejection fraction; NA, not available; RCT, randomized controlled trial.

### Primary clinical outcomes

**Figure 3:**
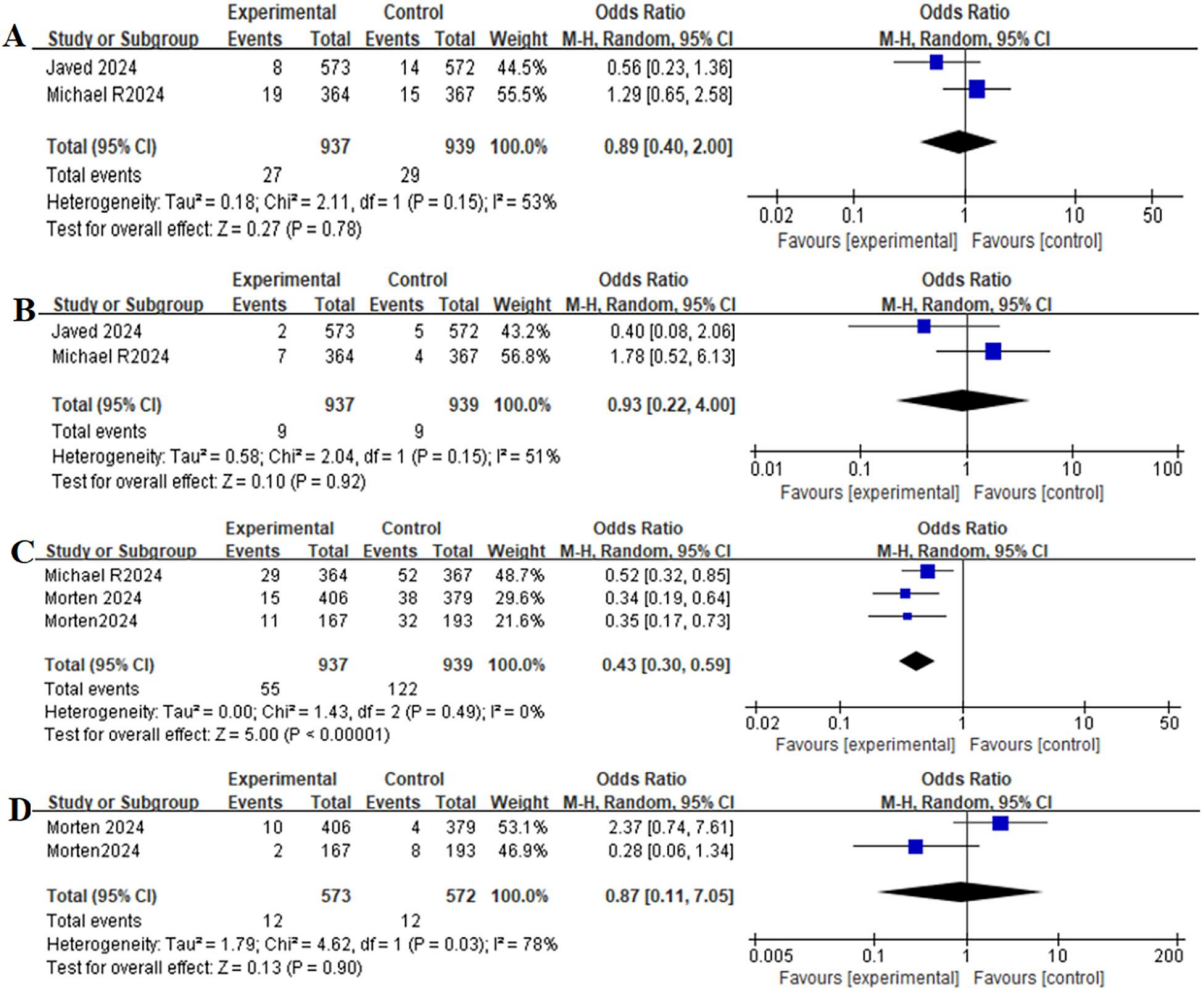
Forest plot of the meta-analysis for all-cause mortality-**(A)**, death from cardiovascular causes-**(B)**, worsening heart-failure events-**(C)**, and gastrointestinal adverse events-**(D)** (Experimental group: Patients received GLP-1 receptor agonists; Control group: Patients received Placebo).

Table 2 provides a brief overview of the Primary clinical outcomes. The primary clinical outcomes of patients receiving GLP-1RAs as treatment for obese patients with CHF were as follows: all-cause mortality (OR = 0.89, 95% CI:0.40–2.00, *p* = 0.78) (Figure 3A), cardiovascular mortality (OR = 0.93,95% CI: 0.22–4.00, *p* = 0.92) (Figure 3B)and worsening heart failure events (OR = 0.43,95% CI: 0.30–0.59, *p* < 0.00001) (Figure 3C).

**Table 2:** The results of the meta-analysis for all-cause mortality,cardiovascular mortality, worsening heart-failure events, gastrointestinal adverse events, serious adverse events, change in body weight, change in the KCCQ-CSS,change in the 6-minute walk distance,change in the BNP level,change in the hs-CRP level,and change in the LVEF level.

Outcomes	OR	MD	LCI	UCI	Overall effect size	Heterogeneity	
						I^2^	P
All-cause mortality	0.89	NA	0.40	2.00	0.78	53%	0.15
Cardiovascular mortality	0.93	NA	0.22	4.00	0.92	51%	0.15
Worsening heart failure events	0.43	NA	0.30	0.59	<0.00001	0%	0.49
Gastrointestinal adverse events	0.87	NA	0.11	7.05	0.90	78%	0.03
Serious adverse events	0.63	NA	0.37	1.08	0.09	83%	0.003
Change in body weight	NA	−7.90	−15.44	−0.35	0.04	99%	<0.00001
Change in the KCCQ-CSS	NA	6.81	6.62	6.99	<0.00001	0%	0.51
Change in the 6-minute walk distance	NA	15.91	15.36	16.47	<0.00001	0%	0.64
Change in the BNP level	NA	−0.13	−0.21	−0.05	0.001	49%	0.12
Change in the hs-CRP level	NA	−16.61	−48.53	15.31	0.31	100%	<0.00001
Change in the LVEF level	NA	−0.91	−2.12	0.29	0.14	0%	0.68

KCCQ-CSS, Kansas City Cardiomyopathy Questionnaire Clinical Summary Score; BNP, B-type natriuretic peptide; hs-CRP, high-sensitivity C-Reactive Protein; LVEF, left ventricular ejection fraction;OR, Odds Ratio; MD, Mean Difference; LCI, lower confidence interval; UCI, upper confidence interval;NA, not available.

### Safety outcomes

**Figure 4:**
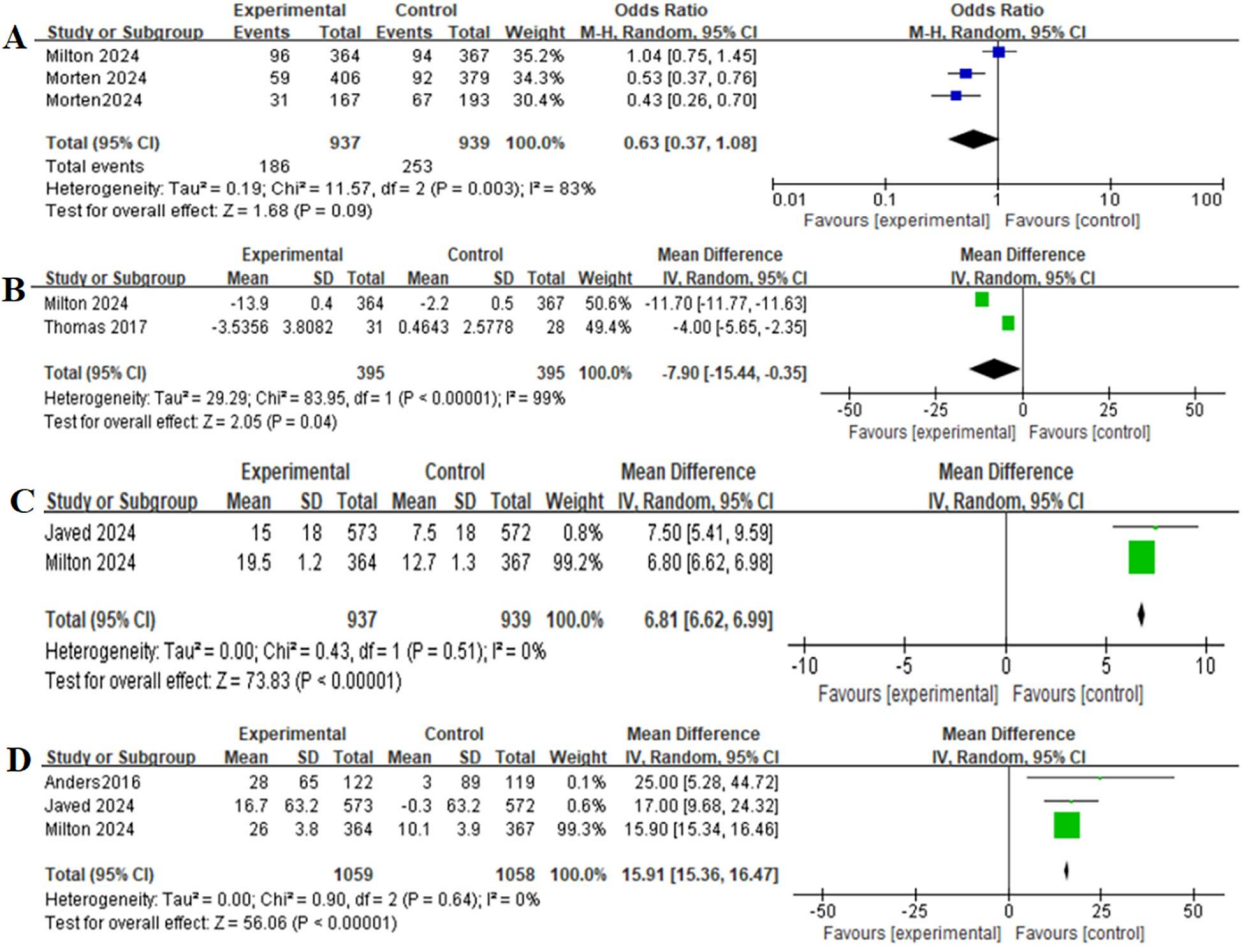
Forest plot of the meta-analysis for serious adverse events-**(A)**, change in body weight-**(B)**, change in the KCCQ-CSS-**(C)**, and change in the 6-minute walk distance-**(D)** (experimental group: patients received GLP-1 receptor agonists; control group: patients received placebo or glimepiride).KCCQ-CSS, Kansas city cardiomyopathy questionnaire clinical summary score.

Table 2 provides a brief overview of the safety outcomes. Safety outcomes of patients receiving GLP-1RAs as treatment for obese patients with CHF were as follows: gastrointestinal adverse event (OR = 0.87,95% CI: 0.11–7.05, *p* = 0.90) (Figure 3D), serious adverse event (OR = 0.63,95% CI: 0.37–1.08, *p* = 0.09) (Figure 4A).

### Secondary outcomes

**Figure 5:**
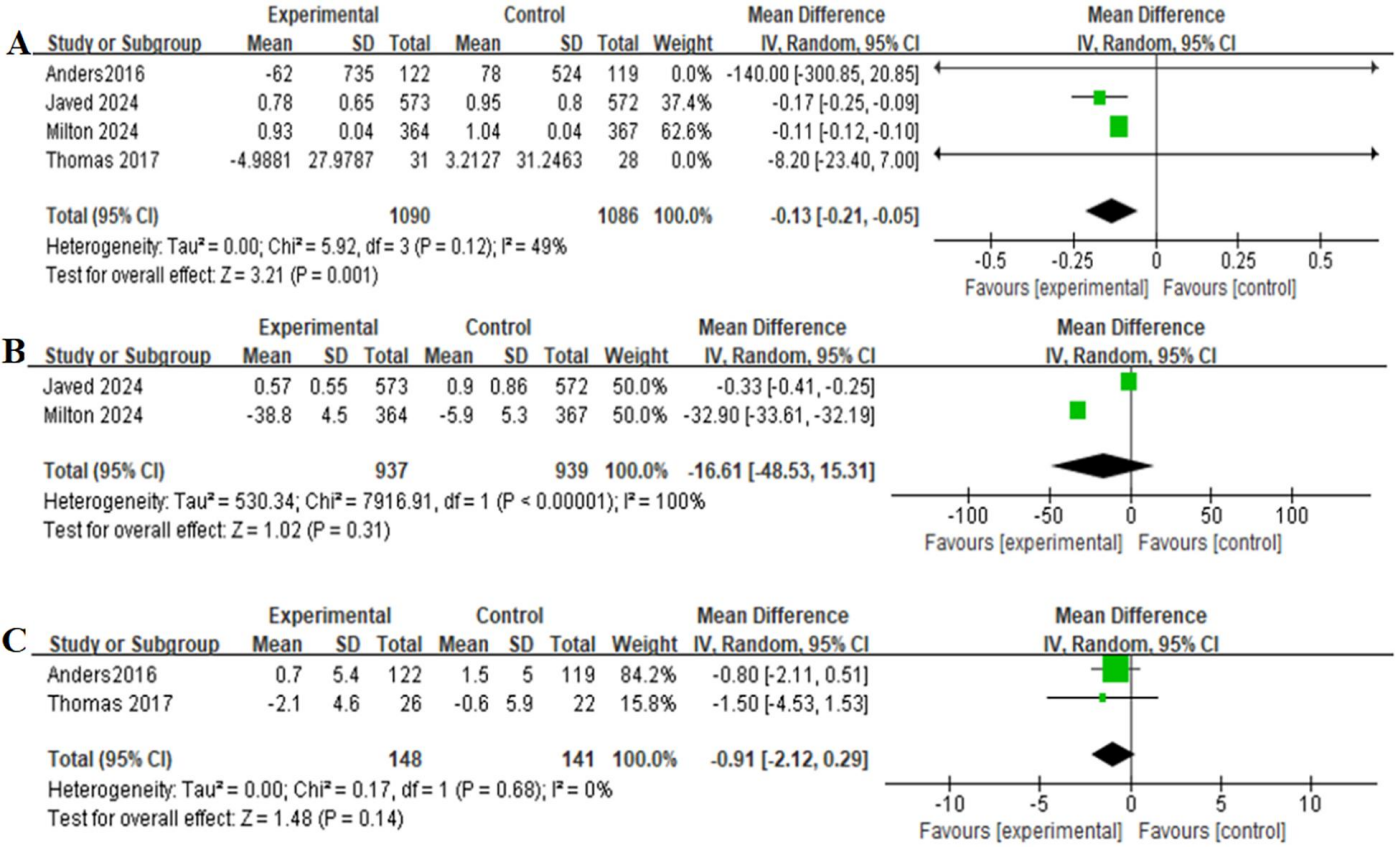
Forest plot of the meta-analysis for change in the BNP level-**(A)**, change in the hs-CRP level-**(B)**, and change in the LVEF level-**(C)** (experimental group: patients received GLP-1 receptor agonists; control group: patients received placebo or glimepiride).BNP, B-type natriuretic peptide; hs-CRP, high-sensitivity C-reactive protein; LVEF, left ventricular ejection fraction.

Table 2 provides a brief overview of the secondary outcomes. Functional/biomarker/Imaging outcomes of patients receiving GLP-1RAs as treatment for obese patients with CHF were as follows:change in body weight (MD = −7.90,95% CI: −15.44 to −0.35, *p* = 0.04) (Figure 4B), change in the KCCQ-CSS (MD = 6.81,95% CI:6.62–6.99, *p* < 0.00001) (Figure 4C), change in the 6-minute walk distance (MD = 15.91,95% CI: 15.36–16.47,*p* < 0.00001) (Figure 4D), change in the BNP level (MD = −0.13,95% CI: −0.21 to−0.05,*p* = 0.001) (Figure 5A), change in the hs-CRP level (MD = −16.61, 95% CI:−48.53–15.31,*p* = 0.31) (Figure 5B), and change in the LVEF level (MD = −0.91,95% CI: −2.12–0.29, *p* = 0.14) (Figure 5C).

## Discussion

The results of this meta-analysis provide important insights into the efficacy and safety of GLP-1RAs in the treatment of obese CHF patients. Our results show that GLP-1RAs such as Liraglutide, Semaglutide, and Tirzepatide showed significant improvements in key clinical outcomes such as weight loss, improved KCCQ-CSS, increased 6-min walk distance, and decreased BNP levels. In terms of primary clinical outcomes, GLP-1RAs significantly reduced the risk of worsening heart failure events notably, but no significant changes were observed in all-cause mortality, cardiovascular mortality.

The results showed that GLP-1RAs significantly reduced the risk of worsening heart failure events in the primary clinical outcome (worsening heart failure events: OR = 0.43, 95% CI: 0.30–0.59, *p* < 0.00001), but it had no effect on all-cause mortality and cardiovascular mortality (all-cause mortality: OR = 0.89, 95% CI: 0.40–2.00, *p* = 0.78; cardiovascular mortality: OR = 0.93, 95% CI: 0.22–4.00, *p* = 0.92). Shaylee and Arden (24) reported in their meta-analysis that GLP-1RAs significantly reduced all-cause mortality in patients with type 2 diabetes mellitus at high cardiovascular risk, but showed no significant effect on cardiovascular mortality. Arunkumar et al. (25), in a large population-matched cohort study, found that there was no significant difference between GLP-1RAs and SGLT2 inhibitors in reducing all-cause mortality in individuals with non-alcoholic fatty liver disease (NAFLD) and type 2 diabetes. Jo ˜ ao et al. (26) concluded in a analysis that liraglutide may increase the risk of adverse cardiovascular effects in patients with HFrEF. Other studies support the potential benefit of GLP-1RAs in reducing all-cause mortality, cardiovascular mortality, and worsening heart failure events (27–29). Future studies with larger samples and longer follow-up times are needed to further validate the effects of GLP-1RAs on all-cause mortality, cardiovascular mortality, and worsening heart failure events.

In secondary outcomes, GLP-1RAs significantly reduced patients' body weight (MD = −7.90, 95% CI: −15.44 to −0.35, *p* = 0.04), which is consistent with previous studies. Daniel et al. (30) found that among obese adolescents, a weekly 2.4 mg dose of semaglutide treatment plus a lifestyle intervention resulted in a greater reduction in BMI compared to lifestyle intervention alone in a double-blind, parallel-group, randomized, placebo-controlled trial. Similarly, Xie et al. (31) reported that GLP-1RAs exhibited significant glucose-lowering and weight-loss effects in patients with type 2 diabetes mellitus combined with a high BMI.GLP-1 agonists work by mimicking the physiological effects of GLP-1, delaying gastric emptying, increasing satiety, and reducing food intake; at the same time, they can directly stimulate the satiety centres in the hindbrain and hypothalamus, further suppressing appetite (32). In addition, liraglutide and semaglutide have been approved for weight loss treatment in overweight patients with comorbidities or obesity (33). GLP-1RAs also significantly improved KCCQ-CSS (MD = 6.81, 95% CI: 6.62–6.99, *p* < 0.00001) and 6-minute walk distance (MD = 15.91, 95% CI: 15.36–16.47, *p* < 0.00001).The significant increase in KCCQ-CSS and 6-min walking distance suggests that GLP-1RAs may improve cardiac function, reduce heart failure symptoms, and increase exercise tolerance in patients.GLP-1RAs may exert these benefits by reducing the generation of reactive oxygen species (ROS), decreasing systemic inflammation, and improving diastolic function (e. g., reducing diastolic filling pressure and ventricular load) (34). Amrit et al. (35) demonstrated that liraglutide not only improved myocardial perfusion and energy metabolism but also enhanced exercise tolerance (assessed by 6-minute walking distance) in patients compared to pioglitazone in their randomised crossover single-centre study.Melanie et al. (36) found that semaglutide significantly improved patients' symptoms of heart failure (e.g., dyspnea, fatigue) and physical function limitations (e.g., decreased mobility) in a prespecified analysis. In terms of biomarkers, GLP-1RAs significantly reduced BNP levels (MD = −0.13, 95% CI: −0.21 to −0.05, *p* = 0.001), which aligns with the results of a meta-analysis by Angelo et al., showing that GLP-1RAs significantly reduced N-terminal pro-BNP levels (37). However, GLP-1RAs did not improve hs-CRP levels (MD = −16.61, 95% CI: −48.53–15.31, *p* = 0.31), although Mohsen et al. demonstrated that GLP-1RAs significantly reduced serum CRP concentrations in patients with type 2 diabetes in their meta-analysis (38). The observed result of this study may be affected by high heterogeneity (I^2^ = 100%), and more large sample, high quality studies are needed for further verification. In addition, the improvement of LVEF by GLP-1RAs was not significant (MD = −0.91, 95% CI: −2.12–0.29, *p* = 0.14).Arif et al. (39) showed that GLP 1-RAs improved LVEF, significantly benefiting the management of HFpEF in patients with T2DM in a meta-analysis. Zhang et al. (40) concluded that GLP-1RAs may improve left ventricular function in HF patients in a single-center, prospective, interventional study.This result observed in the present study may be related to the specific characteristics of the study population and the relatively small sample size. Obese patients with CHF have complex pathophysiological mechanisms, often associated with metabolic disorders, chronic inflammation, and myocardial fibrosis, and these factors may influence the effects of GLP-1RAs on LVEF. Furthermore, it is crucial to recognize the limitations of LVEF as a cardiac function assessment tool. While primarily measuring myocardial contractility, LVEF shows limited sensitivity to subtle changes in diastolic function and ventricular remodeling, particularly in HFpEF where LVEF typically remains within normal ranges. The stability of LVEF suggests that the clinical benefits of GLP-1 receptor agonists (GLP-1RAs) may stem from non-positive inotropic mechanisms, such as systemic hemodynamic effects, improved myocardial metabolism, anti-inflammatory actions, and anti-fibrotic properties. Therefore, the lack of significant changes in LVEF does not diminish the potential value of GLP-1RAs in heart failure treatment, but rather distinguishes their mechanism of action from that of positive inotropic agents.

In the safety outcomes, GLP-1RAs had no significant effect on gastrointestinal adverse events (OR = 0.87, 95% CI: 0.11–7.05, *p* = 0.90). Nevertheless, Jamy et al. (41) noted that GLP-1RAs may increase the risk of gastrointestinal adverse events, but these adverse events are usually mild to moderate and transient. Future studies with larger samples and longer follow-up times are needed to further clarify the gastrointestinal safety of GLP-1RAs. On the other hand, there was no significant effect of GLP-1RAs group on the incidence of serious adverse events(OR = 0.63, 95% CI: 0.37–1.08, *p* = 0.09).Thomas et al. (42) noted that both tirzepatide and semaglutide did not increase the risk of serious adverse events in adults with type 2 diabetes in a network meta-analysis.This result of this study needs to be further confirmed in the future.

Our study outcomes differ from previous meta-analyses in certain endpoints (e.g., all-cause mortality, cardiovascular mortality, hs-CRP levels). We will delve into the potential reasons for these discrepancies. First, our chronic heart failure analysis included patients with various cardiac conditions, whereas other studies focused exclusively on HFpEF or HFrEF patients. This fundamental shift in patient composition constitutes the core reason for the outcome differences. Second, previous Meta-analyses likely relied more heavily on evidence from first-generation GLP-1 receptor agonists (e.g., liraglutide), while our analysis incorporates a higher proportion of newer, more potent drugs (e.g., semaglutide). Variations in drug molecular structures, half-lives, and receptor affinities may lead to differences in therapeutic efficacy and anti-inflammatory potency. Given the high heterogeneity in hs-CRP measurements (I^2^ = 100%), it's not surprising that our pooled estimates differ from previous Meta-analyses. These differences likely reflect significant variations in measurement methods, laboratory standards, or baseline inflammatory status rather than genuine differences in drug efficacy.

The study has its advantage. This study sets up a rich set of evaluation indicators, focusing not only on key primary outcomes such as all-cause mortality, cardiovascular mortality, and worsening heart failure events, but also on secondary outcomes like weight changes. It also considers safety metrics such as gastrointestinal adverse reactions and serious adverse events, providing a comprehensive assessment of the application of GLP-1RAs in obese CHF patients. Furthermore, it targets the specific group of obese patients with chronic heart failure, whose conditions are complex and who face unique treatment needs and challenges, making the research highly targeted.

The present meta-analysis has several limitations. First, the included studies varied in population characteristics, drug type、 dose, and follow-up time, leading to high heterogeneity of results (e.g., I^2^ = 100% for hs-CRP levels), which may affect the universality and reliability of the results. The heterogeneity of hs-CRP reached 100%, indicating that differences among studies almost entirely determined the variation in results rather than sampling errors. We speculate this may stem from three aspects: First, methodological heterogeneity. Although all studies used “hs-CRP”, variations in testing platforms and inconsistent quality control protocols across laboratories could introduce systematic measurement bias. Second, clinical heterogeneity. As a highly sensitive but nonspecific inflammatory marker, hs-CRP measurements may vary significantly between study participants' baseline conditions. Some studies permitted use of other anti-inflammatory drugs, which might confound the true effects of interventions on hs-CRP levels. Third, intervention impacts are not universal but heavily dependent on specific study contexts and population characteristics. Future research should define more homogeneous subgroups for analysis. Gastrointestinal adverse events, being subjective outcomes, exhibit complex heterogeneity due to inconsistent definitions across studies. Significant variations in baseline gastrointestinal health status and tolerance to intervention components lead to substantial response differences. The moderate-to-high heterogeneity (I^2^ = 78%) highlights that intervention-related gastrointestinal risks are not fixed, necessitating individualized risk assessment. In conclusion, these specific highly heterogeneous outcomes strongly suggest that the impact of our interventions on these indicators is context-dependent. Although the random effects model attempts to account for study-to-study variation, it does not eliminate the heterogeneity itself. Second, the small sample size of some studies, such as the study by Thomas et al. (2017) included only 62 patients, may not be sufficient to detect significant differences (22). In addition, no subgroup analysis (ie by drug type, dose) was performed due to the limited number of included studies, which limits the exploration of potential differences in different subgroups. Future research should prioritize this aspect, and can conduct head-to-head randomized controlled trials (RCTs), perform network meta-analysis (NMA), accumulate more studies for dose-response meta-analysis, and carry out meta-analysis using individual participant data (IPD). Third, the short duration of follow-up in most studies (e.g., 52 weeks) makes it difficult to assess the long-term effects and safety of GLP-1RAs, for example, whether weight regain will occur after discontinuation or the long-term effects on cardiovascular events are not known.Future studies should extend the follow-up time of existing RCTs, conduct longer prospective long-term RCTs, and perform patient-level Meta analysis. Furthermore,due to limited number of studies, subgroup analysis by ejection fraction (EF) was not conducted.The response of patients with heart failure in different ejection fraction ranges to treatment may vary. Future studies with larger sample sizes are needed to explore the possible efficacy differences based on the EF status. Finally, it should be noted that this study included patients in the overweight category. Specifically, as presented in Table 1, the mean BMI of one cohort was 28. While this may be perceived as a deviation from the conventional definition of obesity, we contend that it offers a unique and clinically relevant perspective. Grounded in the context of the “obesity paradox,” the applicability of traditional BMI classifications for heart failure patients remains debated. Both overweight and obesity share common pathophysiological underpinnings, such as insulin resistance and features of heart failure mechanisms like volume overload and impaired myocardial metabolism. Consequently, this study aimed to move beyond strict BMI cut-offs to evaluate the therapeutic value of GLP-1RAs not only in a “strictly defined obese” population but also in a “broader spectrum of heart failure patients who, while potentially benefiting from the obesity paradox, might still require active weight management” (encompassing the range from overweight to obesity). We acknowledge that employing broad BMI inclusion criteria may introduce heterogeneity; however, this approach better reflects the complexity of real-world clinical practice and provides more comprehensive evidence on the use of GLP-1RAs in heart failure patients with concomitant metabolic abnormalities.

## Conclusion

In conclusion, GLP-1 RAs represent a promising treatment option for obese patients with CHF. Our findings show that these agents significantly reduce the risk of worsening heart failure events and confer substantial clinical benefits, including improved symptom burden (KCCQ-CSS), greater functional capacity (6-minute walk distance), and reduced levels of BNP, a key heart failure biomarker. These improvements occurred despite the absence of a significant change in LVEF.

## Data Availability

The original contributions presented in the study are included in the article/Supplementary Material, further inquiries can be directed to the corresponding author/s.
