# Supplementary material for: Efficacy and safety of GLP-1 receptor agonists in the treatment of obese patients with chronic heart failure: a meta-analysis
Source: Front Cardiovasc Med. 2025 Oct 3;12:1633114. doi: 10.3389/fcvm.2025.1633114 (PMC12533278; doi:10.3389/fcvm.2025.1633114)
Supplement: Supplementary file 1 [file Datasheet1.pdf]

PubMed		
No.	Query	Results
#1	(((((((((((((chronic heart failure) OR (Cardiac Failure)) OR (Heart Decompensation)) OR (Decompensation, Heart)) OR (Heart Failure, Right-Sided)) OR (Heart Failure, Right Sided)) OR (Right-Sided Heart Failure)) OR (Right Sided Heart Failure)) OR (Myocardial Failure)) OR (Congestive Heart Failure)) OR (Heart Failure, Congestive)) OR (Heart Failure, Left-Sided)) OR (Heart Failure, Left Sided)) OR (Left-Sided Heart Failure)) OR (Left Sided Heart Failure)	374571
#2	(((((Tirzepatide) OR (Semaglutide)) OR (((((((((((Lixisenatide) OR (AVE 0010)) OR (AVE-0010)) OR (AVE0010)) OR (AQVE-10010)) OR (Lyxumia)) OR (Adlyxin)) OR (AVE 010)) OR (AVE-010)) OR (ZP10A peptide)) OR (ZP 10)) OR (ZP-10))) OR (((((Dulaglutide) OR (LY 2189265)) OR (LY-2189265)) OR (LY2189265)) OR (Trulicity))) OR ((((((((((Exenatide) OR (Byetta)) OR (AC 2993)) OR (Bydureon)) OR (Exendin-4)) OR (Ex4 Peptide)) OR (Peptide, Ex4)) OR (Exendin 4)) OR (ITCA 650)) OR (AC 2993 LAR))) OR (((((((Liraglutide) OR (Victoza)) OR (saxena)) OR (NN 2211)) OR (2211, NN)) OR (NN2211)) OR (NN-2211))	26874
#3	#1 AND #2	677

Cochrane library		
No.	Query	Results
#1	(chronic heart failure OR Cardiac Failure OR Heart Decompensation OR Decompensation, Heart OR Heart Failure, Right-Sided OR Heart Failure, Right Sided OR Right-Sided Heart Failure OR Right Sided Heart Failure OR Myocardial Failure OR Congestive Heart Failure OR Heart Failure, Congestive OR Heart Failure, Left-Sided OR Heart Failure, Left Sided OR Left-Sided Heart Failure OR Left Sided Heart Failure):ab,ti,kw	37151
#2	(Tirzepatide OR Semaglutide OR Lixisenatide OR AVE 0010 OR AVE-0010 OR AVE0010 OR AQVE-10010 OR Lyxumia OR Adlyxin OR AVE 010 OR AVE-010 OR ZP10A peptide OR ZP 10 OR ZP-10 OR Dulaglutide OR LY 2189265 OR LY-2189265 OR LY2189265 OR Trulicity OR Exenatide OR Byetta OR AC 2993 OR Bydureon OR Exendin-4 OR Ex4 Peptide OR Peptide, Ex4 OR Exendin 4 OR ITCA 650 OR AC 2993 LAR OR Liraglutide OR Victoza OR saxena OR NN 2211 OR 2211, NN OR NN2211 OR NN-2211):ab,ti,kw	6223

#3	#1 AND #2	206

Embase		
No.	Query	Results
#1	'chronic heart failure':ab,ti OR 'Cardiac Failure':ab,ti OR 'Heart Decompensation':ab,ti OR 'Decompensation, Heart':ab,ti OR 'Heart Failure, Right-Sided':ab,ti OR 'Heart Failure, Right Sided':ab,ti OR 'Right-Sided Heart Failure':ab,ti OR 'Right Sided Heart Failure ':ab,ti OR 'Myocardial Failure':ab,ti OR 'Congestive Heart Failure':ab,ti OR 'Heart Failure, Congestive':ab,ti OR 'Heart Failure, Left-Sided':ab,ti OR 'Heart Failure, Left Sided':ab,ti OR 'Left-Sided Heart Failure':ab,ti OR 'Left Sided Heart Failure':ab,ti	116763
#2	'Tirzepatide':ab,ti OR 'Semaglutide':ab,ti OR 'Lixisenatide':ab,ti OR 'AVE 0010':ab,ti OR 'AVE-0010':ab,ti OR 'AVE0010':ab,ti OR 'AQVE-10010':ab,ti OR 'Lyxumia':ab,ti OR 'Adlyxin':ab,ti OR 'AVE 010':ab,ti OR 'AVE-010':ab,ti OR 'ZP10A peptide':ab,ti OR 'ZP 10':ab,ti OR 'ZP-10':ab,ti OR 'Dulaglutide':ab,ti OR 'LY 2189265':ab,ti OR 'LY-2189265':ab,ti OR 'LY2189265':ab,ti OR 'Trulicity':ab,ti OR 'Exenatide':ab,ti OR 'Byetta':ab,ti OR 'AC 2993':ab,ti OR 'Bydureon':ab,ti OR 'Exendin-4':ab,ti OR 'Ex4 Peptide':ab,ti OR 'Peptide, Ex4':ab,ti OR 'Exendin 4':ab,ti OR 'ITCA 650':ab,ti OR 'AC 2993 LAR':ab,ti OR 'Liraglutide':ab,ti OR 'Victoza':ab,ti OR 'saxena':ab,ti OR 'NN 2211':ab,ti OR '2211, NN':ab,ti OR 'NN2211':ab,ti OR 'NN-2211':ab,ti	19353
#3	#1 AND #2	65
